# Flux prediction using artificial neural network (ANN) for the upper part of glycolysis

**DOI:** 10.1371/journal.pone.0216178

**Published:** 2019-05-08

**Authors:** Anamya Ajjolli Nagaraja, Nicolas Fontaine, Mathieu Delsaut, Philippe Charton, Cedric Damour, Bernard Offmann, Brigitte Grondin-Perez, Frederic Cadet

**Affiliations:** 1 LE2P, Laboratory of Energy, Electronics and Processes EA 4079, Faculty of Sciences and Technology, University of La Reunion, France; 2 PEACCEL, n°6 Square Albin Cachot, Paris, France; 3 DSIMB, INSERM, UMR S-1134, Laboratory of ExcellenceLABEX GR, Faculty of Sciences and Technology, University of La Reunion & University Paris Diderot, Paris, France; 4 Université de Nantes, Unité Fonctionnalité et Ingénierie des Protéines (UFIP), UMR 6286 CNRS, UFR Sciences et Techniques, chemin de la Houssinière, France; Universite Paris-Sud, FRANCE

## Abstract

The selection of optimal enzyme concentration in multienzyme cascade reactions for the highest product yield in practice is very expensive and time-consuming process. The modelling of biological pathways is a difficult process because of the complexity of the system. The mathematical modelling of the system using an analytical approach depends on the many parameters of enzymes which rely on tedious and expensive experiments. The artificial neural network (ANN) method has been successively applied in different fields of science to perform complex functions. In this study, ANN models were trained to predict the flux for the upper part of glycolysis as inferred by NADH consumption, using four enzyme concentrations *i*.*e*., phosphoglucoisomerase, phosphofructokinase, fructose-bisphosphate-aldolase, triose-phosphate-isomerase. Out of three ANN algorithms, the neuralnet package with two activation functions, “logistic” and “tanh” were implemented. The prediction of the flux was very efficient: RMSE and R^2^ were 0.847, 0.93 and 0.804, 0.94 respectively for logistic and tanh functions using a cross validation procedure. This study showed that a systemic approach such as ANN could be used for accurate prediction of the flux through the metabolic pathway. This could help to save a lot of time and costs, particularly from an industrial perspective. The R-code is available at: https://github.com/DSIMB/ANN-Glycolysis-Flux-Prediction.

## Introduction

The emergence of genomics, transcriptomics and proteomics, along with improvements in information technology, helped us to integrate the information, build the mathematical *in silico* model of a biological system and observe its behaviour [1–5]. The integration of different “-omics” data helped us to understand the genetic difference between the phenotypes, to identify the molecular signature [6,7] and use metabolic engineering [8,9] etc. There have been many attempts to model biological systems, like *Saccharomyces cerevisiae* [4,10–12], *Escherichia coli* [13–15], other organisms [3] and many plant metabolic networks for observing and predicting the behaviour of a system using different methods [2,16].

Many different kinds of mathematical models exist to study biological systems [17,18]. Several approaches have been developed to determine or estimate the flux through the metabolic pathway [19–21]. Based on the data and constraints used, the mathematical modelling can be classified into two broad categories [2,16] i.e., kinetic modelling or mechanistic modelling [22–24], and constraint-based or stoichiometric modelling [12,25,26]. The kinetic model defines the reaction mechanism in the system using kinetic parameters to evaluate rate laws. These rate laws are defined from the experiment, assuming that the experimental conditions are similar to *in-vivo* conditions [27]. To build a kinetic model, the system has been made as simple as possible, while retaining system behaviour. The modelling of enzymes like phosphofructokinase could be problematic and might need more parameters than other enzymes [28]. Determining the kinetic parameter is expensive and time consuming; some parameters could be more difficult to measure. Although many enzymatic assays are described in the literature, sometimes it is necessary to modify the assay for new enzymes or to find a new one. In some cases, for example, following enzyme reaction through spectrophotometers or spectrofluorimeters, this is difficult due to no absorption or emission signals [29] linked to the reactants. Most of the available kinetic data are obtained from *in-vitro* studies using purified enzymes which might not represent the exact properties of *in-vivo* enzymes [23]. For example: The V_max_ value measured *in vitro* may not represent the value of an *in vivo* system because of the destruction of enzyme complexes, cellular organisation and the absence of an unknown inhibitor or activator [30,31]. A constraint-based model uses physiochemical constraints like mass balance, thermodynamic constraints, etc., in the modelling, to observe and study the behaviour of the system [25]. There are different methods, like flux balance analysis [32] and metabolic flux analysis [33]. Flux balance analysis is an approach to studying biochemical networks on a genomic scale, which includes all the known metabolite reactions, and the genes that encode for a particular enzyme. The data from genome annotation or existing knowledge is used to construct the network [5,34] and the physicochemical constraints are used to predict the flux distribution, considering that the total product formed must be equal to the total substrate consumed in steady state conditions [32]. This method is used to predict the growth rate [5,32,34,35] or the production of a particular metabolite [36]. Metabolic flux analysis, an experimental based method, allows the quantification of metabolite in the central metabolism using the Carbon-labelled substrate [33,37,38]. The labelled substrate is allowed to distribute over the metabolic network and is measured using NMR [39] or mass spectrometry [32].

Many of the biomolecules like organic acids [40,41], antibiotics [42–44], bioethanol etc. [45,46] have been used in the pharmaceutical and food industries and as energy sources. Biomolecule production is attracting the attention of biologists and industries due to the decrease in non-renewable resources and global warming [47,48]. Synthetic biology and systems biology help to obtain the highest yield of biomolecules from the source [49–51].

Glycolysis is the centre of the metabolic system in all living organisms. It is an anaerobic pathway present in almost all living cells and helps in ATP generation. Glycolysis has been established as the central core for the fermentation. It contributes to the production of different metabolites, like citric acid, succinic acid, amino acids, etc., through pyruvate, the end product of glycolysis [52].

The neural network is an architecture, modelled on the brain, that is organized with neurons and synapses present in a structure of nodes (formal neuron) connected together [53,54]. Each numerical input corresponds to the input layer, and the value to predict (variable to explain) corresponds to the last level, the output layer. Between those two layers, intermediary nodes are present, built specifically and in sufficient number to model the problem; they form the hidden layer. The neural network has been successfully applied in different fields of science such as physics [55,56], environmental science [57–59] and data mining [60,61] for the prediction of different features in the system. The ANN is also core for deep learning [62]. The artificial neural network could be used to predict the product outcome (i.e. flux through the pathway) when combined with Flux balance analysis or other modelling approaches.

The ANN has been used earlier in predicting the fluxes from ^13^C labelling of metabolites in mammalian gluconeogenesis by M.R. Antoniewicz et al. [21]. Three linear regression modelling methods, multiple linear regression (MLR), principal component regression (PCR) and partial least square regression (PLS) were run on simulated data and compared to the ANN. The study showed that ANN, that requires the larger sample (>200) performed better than the other methods for flux prediction using new mass isotopomer data [21].

Due to the challenges in estimating the flux using different methods like constraint-based and kinetic-based modelling approaches [63–66], we developed a simple method using artificial neural networks. This method is based purely on the existing experimental data and hence does not require kinetic parameters as in kinetic modelling and no prior information is required regarding the stoichiometry of the metabolic pathway. In this study, an artificial neural network was built to estimate the flux using enzyme concentrations for the upper part of glycolysis as input. Finding the optimum enzyme concentration—which gives the highest product—through experiments is very tedious and expensive. The neural network approach could be helpful for choosing the optimum enzyme concentrations which enhance the final product concentration without experimental setup, within a short period of time. Experiments were carried out in order to: *i)* assess the structure of the ANN using three different approaches, *ii)* evaluate different activation functions and iii) compare the prediction of flux of NAD^+^ to the fluxes predicted by Fievet & al. (2006).

## Materials and methods

### Principle of artificial neural networks

**Fig 1:**
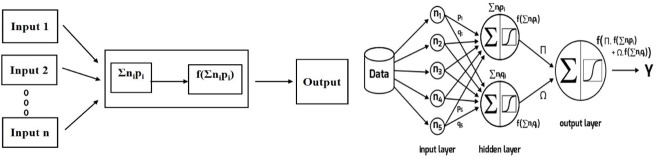
Architecture of artificial neural network.

The base element of ANN is the perceptron, defined in 1958 by Rosenblatt [67]. A combination function computes a value based on the input layer and some weights. This is a weighted sum ∑n_i_p_i_ (observed node) of the n_i_ values in the input layer. To define the output value, a function called activation function is applied to this value. We note n_i_ for the node i, the weight p_i_ corresponds to the connection between node i, the observed node and the activation function f, associated with the observed node (Fig 1).

The structure of an ANN is defined by the number of layers and nodes, by the way they are linked (activation function) and the method to estimate the weights.

### Input for building the ANN model

**Fig 2:**
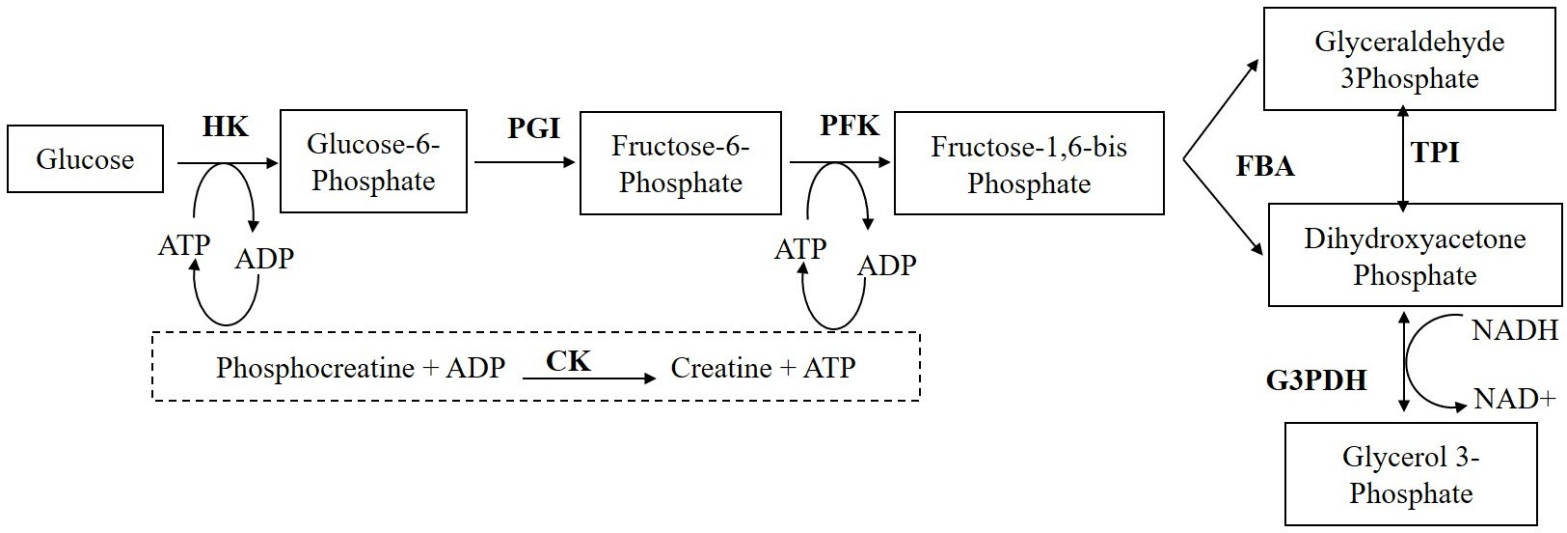
The upper part of glycolysis reconstructed in vitro. HK-hexokinase; PGI-phopshoglucoisomerase; PFK-phosphofructokinase; FBA-fructose bisphosphate aldolase; TPI- triose phosphate isomerase; G3PDH- glycerol-3-phosphate dehydrogenase, CK- Creatine kinase.

The flux measurement data from *in-vitro* reconstructed upper part of glycolysis [20] was used to build the artificial neural network (Fig 2). The input for the ANN model consists of concentrations of enzymes phosphoglucoisomerase (PGI), phosphofructokinase (PFK), fructose bisphosphate aldolase (FBA) and triose phosphate isomerase (TPI) in mg/L, and the output is flux J (μM/s) measured as the NADH consumption by glycerol-3-phosphate dehydrogenase (G3PDH). The flux measured through the upper part of glycolysis is indirect and we assume that most of the NADH in the system is consumed during the measurement. The data has been normalised using min-max method before building the neural network.

### Experimental details

The upper part of glycolysis was reconstructed *in vitro* (Fig 2), with constant concentration of hexokinase andglycerol-3-phosphate dehydrogenase, while the other four enzymes (PGI, PFK, FBA and TPI) concentrations varied. The total enzyme concentration of the four enzymes (PGI, PFK, FBA and TPI) was constant at 101.9 mg/L. The NADH consumption using the glycerol-3-phosphate dehydrogenase is monitored every 2s with Uvikon 850 spectrometer at 390 nm from 60 to 120s. The linear slope of NADH was calculated as the flux through the pathway. The assays were performed in triplicate by Fiévet et al., 2006, at 25°C, by adding 1mM ATP at pH 7.5. Data is given in supplementary information (Table A in S1 File).

### Structure of ANN

The artificial neural network built with a single layer of hidden units [68] using statistical tool R (version 3.4.3) (R Core Team (2013). R: A language and environment for statistical computing. R Foundation for Statistical Computing, Vienna, Austria. http://www.R-project.org/) using three different packages: nnet (version 7.3–12) [69], neuralnet (version 1.33) [70] and RSNNS (version 0.4–10) [71].

The network consists of three layers: a) Input (I), b) Hidden layer (H) and c) Output. These layers are connected by edges or neurons. The weighted sum of neuron inputs is submitted to a function which conditions neuron activation. There is no rule for deciding the number of neuron units in a single hidden layer; to choose the best algorithm out of three (i.e. nnet, neuralnet and RSNNS), we first chose the number of hidden units according to Equation 1 and compared the RMSE (Equation 2) and coefficient of determination (R^2^) (Equation 3) values between the three methods. The algorithm with the lowest RMSE value and highest R^2^value during the leave-one-out cross validation was chosen as an algorithm of interest and the effect of the number of hidden units on RMSE and R^2^ was analysed between 1 to 25 hidden units.

Equation 1: Where Nh is number of hidden units; Ns: number of sampling in training data; Ni: number of input neurons; No: number of output neurons; α: arbitrary scaling factor 2–10.

$$Nh=\frac{Ns}{\alpha \;(Ni+No)}$$

Equation 2: Where RMSE is root mean square error, Yi is ANN predicted values; yi is experimental values; n is number of predictions.

$$RMSE=\sqrt{\frac{\overset{n}{\underset{i=1}{\sum {\left(Y_{i}-y_{i}\right)}^{2}}}}{n}}$$

Equation 3: Where R2 is coefficient of determination; Yi ANN is predicted values; yi is experimental values; n is number of predictions, Ӯ is average of experimental values.

$$R^{2}=1-\frac{\sum_{i}(y_{i}-Y_{i}{)}^{2}}{\sum_{i}(y_{i}-\bar{y}{)}^{2}}\;\mathrm{where},\;\bar{y}=\frac{\sum_{i=1}^{n}y_{i}}{n}$$

## Results and discussion

The three-neural network algorithms: nnet [69], neuralnet [70] and RSNNS [71] are built with a hidden number of units ranging from 9 to12, as shown in Equation 1. The RMSE and coefficient of determination were compared between algorithms during leave-one-out cross validation. Out of three algorithms, the neuralnet performed better than the other two (Table B in S1 File), allowing the option of choosing two different activation functions *i*.*e*., logistic (sigmoidal) and tanh (Equation 4 and Equation 5 respectively).

Equation 4: logistic activation function.

$$Logistic(x)=\frac{1}{1+e^{-x}}$$

Equation 5: tanh activation function.

$$tanh(x)=\frac{2}{1+e^{-2x}}-1$$

**Fig 3:**
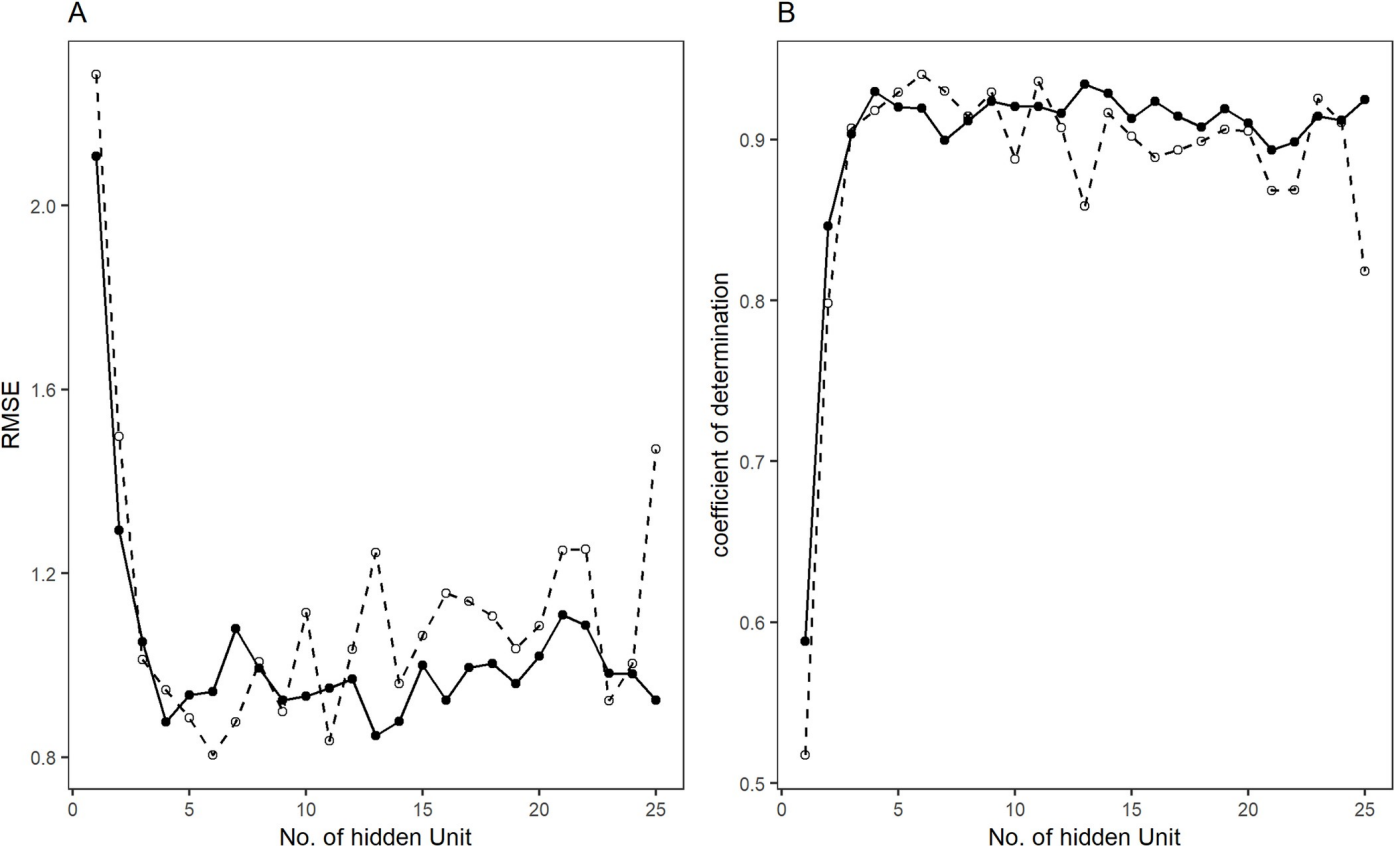
Effect of the number of hidden units on RMSE (A) and coefficient of determination (B) in activation function logistic (filled circle, solid line) and tanh (open circle and dotted lines).

Using the neuralnet model, with “logistic” and “tanh” activation functions, the effect of the number of hidden units on RMSE and R^2^ was studied (Fig 3) with a leave-one-out-cross-validation procedure (Table C in S1 File). The logistic function with 13 hidden units gives a RMSE of 0.847, R^2^ of 0.93 and tanh function RMSE of 0.804, and R^2^ of 0.94 with 6 hidden units. The R-script to build the ANN with leave-one-out cross validation is given in R-Scripts A in S1 File.

**Fig 4:**
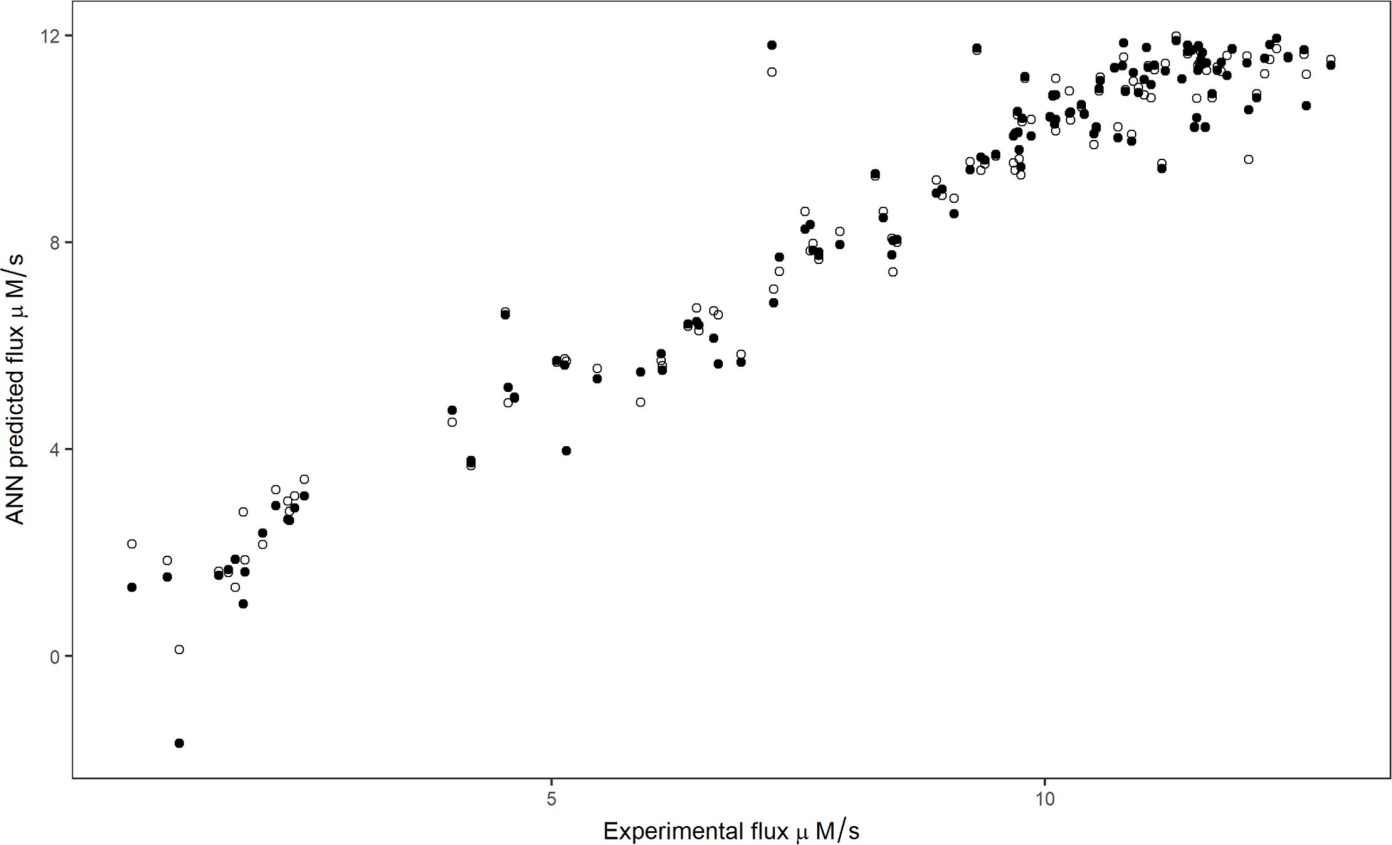
Relationship between flux predicted by leave-one-outcross-validation and experimental flux. Filled and open circles represent logistic and tanh activation functions respectively.

**Fig 5:**
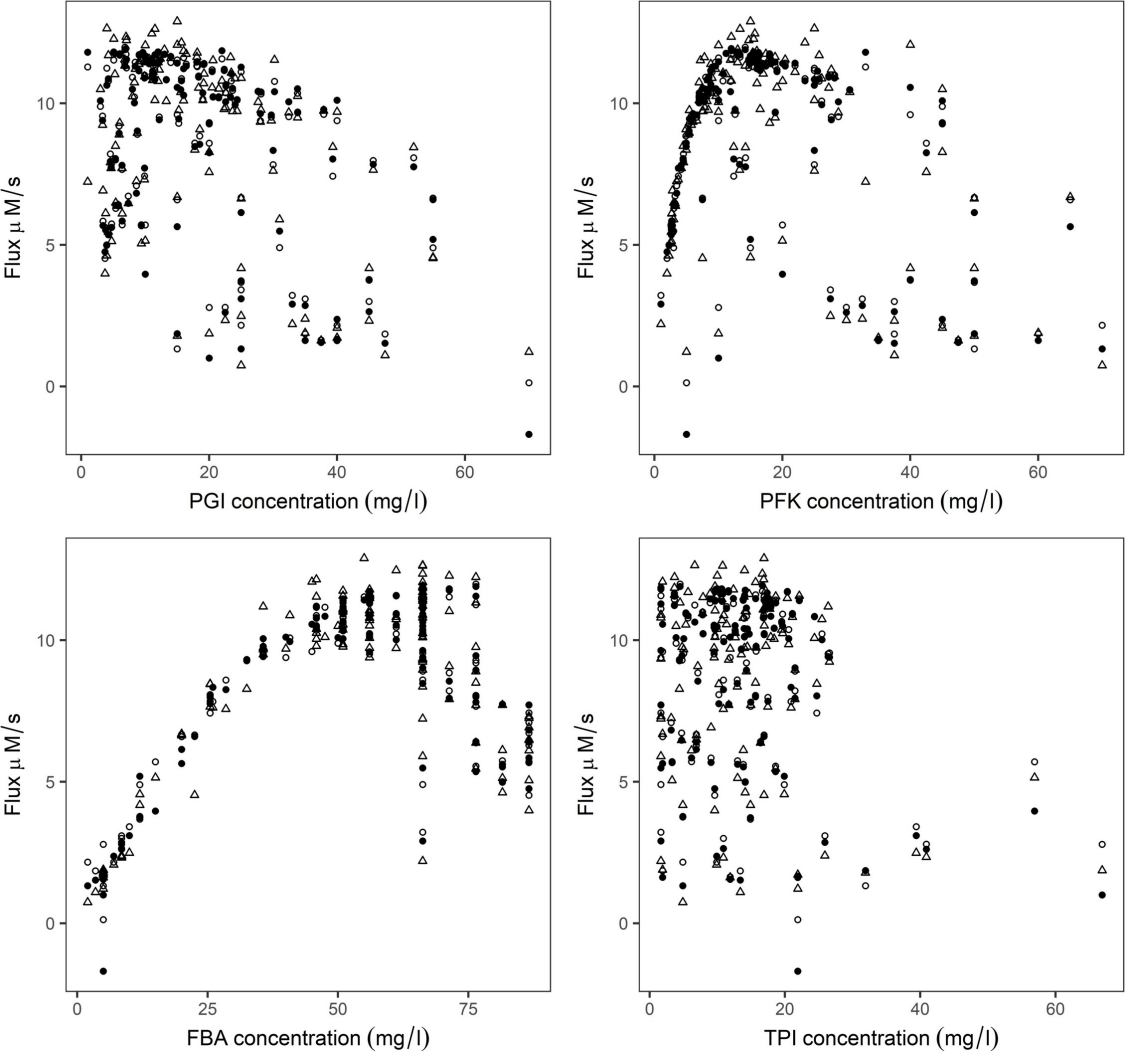
Relationship between the individual enzyme concentration with experimental and ANN predicted flux. Filled circle and open circle are enzyme concentration vs predicted flux with logistic and tanh activation functions respectively, open triangles represent the experiment.

Experimental flux was compared with the ANN predicted flux by leave-one-outcross-validation procedure, using chosen hidden units with logistic and tanh function (Fig 4). The effects of enzyme concentrations on the predicted flux and experimental flux were compared, and found to follow a similar trend (Fig 5).

During the cross validation of the neural network model, a negative flux value was predicted for one combination of enzymes (Table 1: Index-3). This is because, during a leave-one-out procedure (LOOcv), one combination of the four enzymes is not included in the model training and has to be predicted. The negative value shows the poor ability of the ANN model to predict the outliers, i.e. a combination that is not close (in terms of PGI, PFK, FBA and TPI concentrations) to those included in the training data set.

**Table 1:** Comparison of flux values (in *μ*M/S) between observed flux (J_Exp_), J.B Fievet (J_Fievet_)and ANN predicted flux with activation functions logistic (J _{ANN: logistic}_) and tanh (J _{ANN: tanh}*)*_ and the standard deviation of observed flux (J_SD_).

Index	J_Exp_	J_SD_	J_Fievet_	J_{Fievet: Corrected}_	J_{ANN: logistic}_	J _{ANN: tanh}_	Difference_[J_Exp_ : J_Fievet_]	Difference_[J_Exp_ : J_{Fievet:Corrected}_]	Difference_[J_Exp_ : J_{ANN:logistic}_]	Difference_[J_Exp_ : J_{ANN:tanh}_]
1	0.74	0.08	1.14	0.83	1.33	2.16	0.4	0.09	0.59	1.42
2	1.1	0.03	1.97	1.43	1.53	1.85	0.87	0.33	0.43	0.75
3	1.22	0.08	2.44	1.77	-1.69	0.13	1.22	0.55	2.91	1.09
4	1.62	0.05	2.79	2.02	1.56	1.64	1.17	0.4	0.06	0.02
5	1.72	0.02	2.78	2.01	1.66	1.62	1.06	0.29	0.06	0.1
6	1.79	0	2.76	2	1.86	1.32	0.97	0.21	0.07	0.47
7	1.87	0.04	2.6	1.88	1	2.79	0.73	0.01	0.87	0.92
8	1.89	0.01	2.8	2.03	1.62	1.85	0.91	0.14	0.27	0.04
9	2.07	0.12	3.86	2.8	2.37	2.16	1.79	0.73	0.3	0.09
10	2.2	0.06	3.08	2.23	2.91	3.22	0.88	0.03	0.71	1.02
11	2.32	0.06	4.63	3.36	2.64	3	2.31	1.04	0.32	0.68
12	2.34	0.1	4.54	3.29	2.62	2.79	2.2	0.95	0.28	0.45
13	2.39	0.21	4.59	3.33	2.86	3.09	2.2	0.94	0.47	0.7
14	2.49	0.07	5.26	3.81	3.1	3.41	2.77	1.32	0.61	0.92
15	3.99	0.13	4.9	3.55	4.76	4.52	0.91	0.44	0.77	0.53
16	4.18	0.22	6.4	4.64	3.73	3.68	2.22	0.46	0.45	0.5
17	4.18	0.15	6.43	4.66	3.78	3.75	2.25	0.48	0.4	0.43
18	4.53	0.65	8.4	6.09	6.6	6.65	3.87	1.56	2.07	2.12
19	4.56	0.06	5.98	4.33	5.2	4.9	1.42	0.23	0.64	0.34
20	4.62	0.06	5.52	4	5	4.99	0.9	0.62	0.38	0.37
21	5.05	0.13	6.77	4.91	5.71	5.68	1.72	0.14	0.66	0.63
22	5.13	0.19	6.27	4.54	5.62	5.74	1.14	0.59	0.49	0.61
23	5.15	0.26	6.98	5.06	3.97	5.7	1.83	0.09	1.18	0.55
24	5.46	0.1	6.09	4.41	5.36	5.55	0.63	1.05	0.1	0.09
25	5.9	0.03	7.75	5.62	5.49	4.9	1.85	0.28	0.41	1
26	6.11	0.15	6.72	4.87	5.84	5.71	0.61	1.24	0.27	0.4
27	6.12	0.12	6.17	4.47	5.53	5.61	0.05	1.65	0.59	0.51
28	6.38	0.29	7.51	5.44	6.42	6.38	1.13	0.94	0.04	0
29	6.47	0.08	7.77	5.63	6.47	6.72	1.3	0.84	0	0.25
30	6.49	0.09	7.09	5.14	6.4	6.29	0.6	1.35	0.09	0.2
31	6.64	0.1	10.19	7.38	6.14	6.67	3.55	0.74	0.5	0.03
32	6.69	0.11	9.95	7.21	5.64	6.59	3.26	0.52	1.05	0.1
33	6.92	0.24	6.2	4.49	5.68	5.84	0.72	2.43	1.24	1.08
34	7.23	0.01	6.3	4.57	11.8	11.29	0.93	2.66	4.57	4.06
35	7.25	0.11	8.36	6.06	6.83	7.09	1.11	1.19	0.42	0.16
36	7.31	0.04	8.92	6.46	7.72	7.44	1.61	0.85	0.41	0.13
37	7.57	0.65	13.45	9.75	8.26	8.59	5.88	2.18	0.69	1.02
38	7.62	0.15	12.05	8.73	8.34	7.83	4.43	1.11	0.72	0.21
39	7.65	0.32	10.72	7.77	7.84	7.98	3.07	0.12	0.19	0.33
40	7.71	0.34	8.27	5.99	7.74	7.74	0.56	1.72	0.03	0.03
41	7.71	0.11	8.86	6.42	7.81	7.67	1.15	1.29	0.1	0.04
42	7.92	0.11	8.59	6.22	7.95	8.21	0.67	1.7	0.03	0.29
43	8.28	0.33	15.02	10.88	9.32	9.28	6.74	2.6	1.04	1
44	8.36	0.15	10.79	7.82	8.48	8.6	2.43	0.54	0.12	0.24
45	8.45	0.23	10.92	7.91	7.76	8.08	2.47	0.54	0.69	0.37
46	8.46	0.11	10.49	7.6	8.03	7.43	2.03	0.86	0.43	1.03
47	8.5	0.09	8.97	6.5	8.05	8	0.47	2	0.45	0.5
48	8.9	0.06	10.03	7.27	8.94	9.2	1.13	1.63	0.04	0.3
49	8.96	0.08	10.55	7.64	9.03	8.91	1.59	1.32	0.07	0.05
50	9.08	0.35	10.97	7.95	8.56	8.85	1.89	1.13	0.52	0.23
51	9.24	0.04	8.74	6.33	9.4	9.55	0.5	2.91	0.16	0.31
52	9.31	0.1	15.81	11.46	11.75	11.71	6.5	2.15	2.44	2.4
53	9.35	0.46	12.43	9.01	9.64	9.39	3.08	0.34	0.29	0.04
54	9.39	0.22	12.52	9.07	9.59	9.51	3.13	0.32	0.2	0.12
55	9.5	0.18	14.66	10.62	9.7	9.67	5.16	1.12	0.2	0.17
56	9.68	0.14	15.84	11.48	10.05	9.53	6.16	1.8	0.37	0.15
57	9.7	0.55	13.13	9.51	10.11	9.39	3.43	0.19	0.41	0.31
58	9.72	0.18	13.77	9.98	10.53	10.46	4.05	0.26	0.81	0.74
59	9.73	0.11	13.23	9.59	10.13	10.11	3.5	0.14	0.4	0.38
60	9.74	0.05	13.24	9.59	9.79	9.61	3.5	0.15	0.05	0.13
61	9.76	0.03	11.75	8.51	9.45	9.3	1.99	1.25	0.31	0.46
62	9.77	0.13	13.58	9.84	10.4	10.33	3.81	0.07	0.63	0.56
63	9.8	0.27	16.29	11.8	11.2	11.16	6.49	2	1.4	1.36
64	9.86	0.05	12.94	9.38	10.05	10.38	3.08	0.48	0.19	0.52
65	10.05	0.09	13.89	10.07	10.42	10.43	3.84	0.02	0.37	0.38
66	10.08	0.05	13.11	9.5	10.84	10.83	3.03	0.58	0.76	0.75
67	10.1	0.29	13.13	9.51	10.29	10.29	3.03	0.59	0.19	0.19
68	10.11	0.27	13.34	9.67	10.37	10.15	3.23	0.44	0.26	0.04
69	10.11	0.34	16.89	12.24	10.85	11.17	6.78	2.13	0.74	1.06
70	10.25	0.07	12.73	9.22	10.5	10.92	2.48	1.03	0.25	0.67
71	10.26	0.03	13.82	10.01	10.52	10.36	3.56	0.25	0.26	0.1
72	10.37	0.08	13.68	9.91	10.66	10.62	3.31	0.46	0.29	0.25
73	10.4	0.22	17.96	13.01	10.48	10.47	7.56	2.61	0.08	0.07
74	10.5	0.35	12.1	8.77	10.1	9.89	1.6	1.73	0.4	0.61
75	10.52	0.07	13.05	9.46	10.23	10.2	2.53	1.06	0.29	0.32
76	10.55	0.29	19.26	13.96	10.97	10.93	8.71	3.41	0.42	0.38
77	10.56	0.42	17.95	13.01	11.12	11.19	7.39	2.45	0.56	0.63
78	10.71	0.19	17.85	12.93	11.37	11.37	7.14	2.22	0.66	0.66
79	10.74	0.23	11.69	8.47	10.02	10.23	0.95	2.27	0.72	0.51
80	10.79	0.24	17.82	12.91	11.41	11.41	7.03	2.12	0.62	0.62
81	10.8	n,d,	17.51	12.69	11.86	11.57	6.71	1.89	1.06	0.77
82	10.82	0.19	13.48	9.77	10.91	11.76	2.66	1.05	0.09	0.94
83	10.88	0.3	16.88	12.23	9.96	10.08	6	1.35	0.92	0.8
84	10.9	0.14	15.48	11.22	11.28	11.11	4.58	0.32	0.38	0.21
85	10.95	0.26	18.48	13.39	10.9	11	7.53	2.44	0.05	0.05
86	11.01	0.16	17.59	12.75	11.14	10.84	6.58	1.74	0.13	0.17
87	11.03	0.16	13.48	9.77	11.77	10.22	2.45	1.26	0.74	0.81
88	11.05	0.29	18.2	13.19	11.38	11.41	7.15	2.14	0.33	0.36
89	11.08	0.25	14.92	10.81	11.05	10.79	3.84	0.27	0.03	0.29
90	11.11	0.07	17.06	12.36	11.42	11.33	5.95	1.25	0.31	0.22
91	11.19	0.22	14.36	10.41	9.42	9.52	3.17	0.78	1.77	1.67
92	11.22	0.1	13.93	10.09	11.31	11.46	2.71	1.13	0.09	0.24
93	11.33	0.38	16.17	11.72	11.9	11.98	4.84	0.39	0.57	0.65
94	11.39	0.24	13.61	9.86	11.16	11.15	2.22	1.53	0.23	0.24
95	11.45	0.49	16.86	12.22	11.81	11.69	5.41	0.77	0.36	0.24
96	11.45	0.21	17.14	12.42	11.68	11.65	5.69	0.97	0.23	0.2
97	11.49	0.1	15.97	11.57	11.73	11.71	4.48	0.08	0.24	0.22
98	11.52	0.08	13.61	9.86	10.23	10.22	2.09	1.66	1.29	1.3
99	11.54	0.07	14.93	10.82	10.41	10.78	3.39	0.72	1.13	0.76
100	11.55	0.16	15.71	11.38	11.32	11.42	4.16	0.17	0.23	0.13
101	11.56	0.23	17.45	12.64	11.79	11.8	5.89	1.08	0.23	0.24
102	11.57	0.29	16.13	11.69	11.4	11.46	4.56	0.12	0.17	0.11
103	11.58	0.06	16.85	12.21	11.53	11.67	5.27	0.63	0.05	0.09
104	11.6	0	19.32	14	11.66	11.44	7.72	2.4	0.06	0.16
105	11.63	0.14	13.48	9.77	10.23	10.94	1.85	1.86	1.4	0.69
106	11.64	0.05	18.64	13.51	11.46	11.33	7	1.87	0.18	0.31
107	11.7	0.3	14.94	10.83	10.87	10.79	3.24	0.87	0.83	0.91
108	11.75	0.1	17.04	12.35	11.32	11.39	5.29	0.6	0.43	0.36
109	11.79	0.08	17.5	12.68	11.48	11.3	5.71	0.89	0.31	0.49
110	11.85	0.21	18.97	13.75	11.23	11.61	7.12	1.9	0.62	0.24
111	11.9	0.14	17.09	12.38	11.74	11.73	5.19	0.48	0.16	0.17
112	12.05	0.07	14.68	10.64	11.47	11.61	2.63	1.41	0.58	0.44
113	12.07	0.81	18.22	13.2	10.57	9.6	6.15	1.13	1.5	2.47
114	12.15	0.22	17.11	12.4	10.8	10.87	4.96	0.25	1.35	1.28
115	12.23	0.13	16.53	11.98	11.56	11.25	4.3	0.25	0.67	0.98
116	12.28	0.13	14.77	10.7	11.82	11.53	2.49	1.58	0.46	0.75
117	12.35	0.21	14.61	10.59	11.94	11.74	2.26	1.76	0.41	0.61
118	12.47	0.17	17.1	12.39	11.57	11.58	4.63	0.08	0.9	0.89
119	12.63	0.15	16.91	12.25	11.72	11.63	4.28	0.38	0.91	1
120	12.65	0.21	14.5	10.51	10.64	11.24	1.85	2.14	2.01	1.41
121	12.9	0.53	16.79	12.17	11.42	11.53	3.89	0.73	1.48	1.37
Average difference between observed and predicted							3.32	1.05	0.57	0.57
Standard deviation of the difference between observed and predicted							2.14	0.78	0.63	0.57

The original study by J. B. Fievet et al. developed a model to predict flux. As the authors mentioned in their article, their flux predictor overestimates the observed flux by a constant factor. The predicted flux in their method, has an R^2^ value of 0.86, whereas an ANN approach with logistic function showed an R^2^ value to be 0.93 and in case of tanh activation function, an R^2^ of 0.94, obtained with leave-one-out cross validation, which implies that the ANN approach is more efficient in predicting the flux than the method developed in the Fievet study. The effect of enzyme concentrations on the predicted flux by both methods follows a similar trend.

The difference between actual flux and ANN predicted flux was an average of 0.57 μM/s for logistic and for tanh, with a standard deviation of 0.63 and 0.57 respectively (Table 1), whereas the Fievet & al. study (2006) showed an average of 3.3 and a standard deviation of 2.2 with actual predicted values. Fievet et al. stated that their method overestimates the flux values by a constant factor of 1.38. Hence by dividing the predicted flux values by 1.38, corrected values were obtained. The new average corrected value is 1.04 and standard deviation is 0.78 with the experimental values. This indicates that the ANN method performs better than the method described in the original study. This ANN-based method provides additional degrees of freedom over the method proposed in Fievet & al. (2006). Indeed, the number of degrees of freedom increases with the number of hidden units. This makes it possible to obtain an important advantage regarding the error inherent to the learning phase.

## Conclusion

Kinetic modelling of metabolic pathways is challenging because of the difficulties in estimating the kinetic parameters [29,65], and is sometimes expensive because of the high-cost substrates and technologies involved [72,73], whereas the constraint-based model does not use any kinetic parameters but is efficient enough to predict the flux of metabolites. Choosing the optimum enzyme concentrations for the highest flux could be a challenge when conducting experiments. Using artificial intelligence with available experimental data can help us find a quicker and more cost-effective solution for biological problems.

In this study, a neural network model was built successfully with two different activation functions: i.e., logistic (sigmoidal) and tanh, with RMSE and R^2^ values of 0.847, 0.93 and 0.804, 0.94 respectively. The difference between actual flux and ANN predicted flux was an average of 0.57 for both activation functions. The J. B. Fievet et al. method after the correction has a RMSE of 1.30, with a 1.05 difference between predicted and observed flux, which clearly indicates that the ANN method works better than the other method.

It has not escaped our attention that the artificial neural network model depends on the diversity of the training data, and hence training the model with a maximum of variability in the concentration of enzymes plays a crucial role. The model built in this study might not be enough to extrapolate a model for all other enzyme concentrations. In future, the study will be extended to predict the wide range of flux and the enzyme concentration for a particular flux. Experiments in a wet laboratory will be carried out for an upcoming application paper. Even though further work has to be performed, the artificial neural network approach is a promising method in metabolic pathway modelling and could find its place in metabolic engineering and industrial scale biomolecule synthesis.

## Supporting information

S1 File Table A: Input used to build artificial neural network from Fievet et al., 2016. Table B: Comparision of RMSE and R-squared values during the leave-one-out crossvalidation between neuralnet, nnet and RSNNS algorithm. Table C: RMSE and R-squared for values between the activation functions logistics and tanh during leave-one-out cross validation for the neuralnet method. R-Scripts A: R-script used to obtained the Table 1. The different activation function and number of hidden units are selected using "act.fct = " and "hidden = ".
(DOCX)

## Abbreviations

ANN: artificial neural network
PGI: phosphoglucoisomerase
PFK: 6-phosphofructokinase
FBA: fructose bisphosphate aldolase
TPI: triose phosphate isomerase
RMSE: root mean square error
