# Supplementary material for: Flux prediction using artificial neural network (ANN) for the upper part of glycolysis
Source: PLoS One. 2019 May 8;14(5):e0216178. doi: 10.1371/journal.pone.0216178 (PMC6505829; doi:10.1371/journal.pone.0216178)
Supplement: S1 File — Table A: Input used to build artificial neural network from Fievet et al., 2016. Table B: Comparision of RMSE and R-squared values during the leave-one-out crossvalidation between neuralnet, nnet and RSNNS algorithm. Table C: RMSE and R-squared for values between the activation functions logistics and tanh during leave-one-out cross validation for the neuralnet method. R-Scripts A: R-script used to obtained the Table 1. The different activation function and number of hidden units are selected using "act.fct = " and "hidden = ". (DOCX) [file pone.0216178.s001.docx]

# S1 File

Table A: Input used to build artificial neural network from Fievet et al., 2016.

| **PGI** | **PFK** | **FBA** | **TPI** | **Jpred** | **Jobs** | **S.D.** |
| --- | --- | --- | --- | --- | --- | --- |
| 25 | 70 | 2 | 4.9 | 1.14 | 0.74 | 0.08 |
| 47.5 | 37.5 | 3.5 | 13.4 | 1.97 | 1.1 | 0.03 |
| 70 | 5 | 5 | 21.9 | 2.44 | 1.22 | 0.08 |
| 37.5 | 47.5 | 5 | 11.9 | 2.79 | 1.62 | 0.05 |
| 40 | 35 | 5 | 21.9 | 2.78 | 1.72 | 0.02 |
| 15 | 50 | 5 | 31.9 | 2.76 | 1.79 | 0 |
| 20 | 10 | 5 | 66.9 | 2.6 | 1.87 | 0.04 |
| 35 | 60 | 5 | 1.9 | 2.8 | 1.89 | 0.01 |
| 40 | 45 | 7 | 9.9 | 3.86 | 2.07 | 0.12 |
| 33 | 1 | 66.23 | 1.66 | 3.08 | 2.2 | 0.06 |
| 45 | 37.5 | 8.5 | 10.9 | 4.63 | 2.32 | 0.06 |
| 22.5 | 30 | 8.5 | 40.9 | 4.54 | 2.34 | 0.1 |
| 35 | 32.5 | 8.5 | 25.9 | 4.59 | 2.39 | 0.21 |
| 25 | 27.5 | 10 | 39.4 | 5.26 | 2.49 | 0.07 |
| 3.72 | 1.95 | 86.61 | 9.61 | 4.9 | 3.99 | 0.13 |
| 45 | 40 | 12 | 4.9 | 6.43 | 4.18 | 0.22 |
| 25 | 50 | 12 | 14.9 | 6.4 | 4.18 | 0.15 |
| 55 | 7.5 | 22.5 | 16.9 | 8.4 | 4.53 | 0.65 |
| 55 | 15 | 12 | 19.9 | 5.98 | 4.56 | 0.06 |
| 3.98 | 2.28 | 81.52 | 14.12 | 5.52 | 4.62 | 0.06 |
| 9.4 | 2.58 | 86.61 | 3.31 | 6.77 | 5.05 | 0.13 |
| 4.75 | 2.63 | 81.52 | 13 | 6.27 | 5.13 | 0.19 |
| 10 | 20 | 15 | 56.9 | 6.98 | 5.15 | 0.26 |
| 4.23 | 2.62 | 76.42 | 18.62 | 6.09 | 5.46 | 0.1 |
| 31 | 3 | 66.23 | 1.66 | 7.75 | 5.9 | 0.03 |
| 6.4 | 2.69 | 86.61 | 6.19 | 6.72 | 6.11 | 0.15 |
| 3.81 | 2.71 | 81.52 | 13.85 | 6.17 | 6.12 | 0.12 |
| 5.79 | 3.3 | 76.42 | 16.38 | 7.51 | 6.38 | 0.29 |
| 7.36 | 3.21 | 86.61 | 4.72 | 7.77 | 6.47 | 0.08 |
| 5.38 | 3.01 | 86.61 | 6.9 | 7.09 | 6.49 | 0.09 |
| 25 | 50 | 20 | 6.9 | 10.19 | 6.64 | 0.1 |
| 15 | 65 | 20 | 1.9 | 9.95 | 6.69 | 0.11 |
| 3.4 | 2.81 | 86.61 | 9.08 | 6.2 | 6.92 | 0.24 |
| 1 | 33 | 66.23 | 1.66 | 6.3 | 7.23 | 0.01 |
| 8.62 | 3.47 | 86.61 | 3.19 | 8.36 | 7.25 | 0.11 |
| 9.88 | 3.73 | 86.61 | 1.67 | 8.92 | 7.31 | 0.04 |
| 20 | 42.5 | 28.5 | 10.9 | 13.45 | 7.57 | 0.65 |
| 30 | 25 | 26 | 20.9 | 12.05 | 7.62 | 0.15 |
| 45.65 | 13.3 | 25.47 | 17.47 | 10.72 | 7.65 | 0.32 |
| 6.37 | 4.17 | 76.42 | 14.94 | 8.86 | 7.71 | 0.34 |
| 4.66 | 4.03 | 81.52 | 11.69 | 8.27 | 7.71 | 0.11 |
| 4.56 | 4.53 | 71.33 | 21.48 | 8.59 | 7.92 | 0.11 |
| 20 | 45 | 32.5 | 4.4 | 15.02 | 8.28 | 0.33 |
| 17.75 | 4.96 | 66.23 | 12.95 | 10.79 | 8.36 | 0.15 |
| 51.97 | 14.22 | 25.47 | 10.23 | 10.92 | 8.45 | 0.23 |
| 39.32 | 12.38 | 25.47 | 24.71 | 10.49 | 8.46 | 0.11 |
| 5.35 | 4.48 | 76.42 | 15.65 | 8.97 | 8.5 | 0.09 |
| 5.92 | 5.25 | 76.42 | 14.3 | 10.03 | 8.9 | 0.06 |
| 8.76 | 5.41 | 66.23 | 21.49 | 10.55 | 8.96 | 0.08 |
| 18.53 | 4.91 | 71.33 | 7.12 | 10.97 | 9.08 | 0.35 |
| 3.33 | 5.75 | 66.23 | 26.58 | 8.74 | 9.24 | 0.04 |
| 6 | 18 | 66.23 | 11.66 | 15.81 | 9.31 | 0.1 |
| 28 | 6 | 66.23 | 1.66 | 12.43 | 9.35 | 0.46 |
| 29.71 | 6.62 | 56.04 | 9.53 | 12.52 | 9.39 | 0.22 |
| 33.82 | 18.87 | 35.66 | 13.55 | 14.66 | 9.5 | 0.18 |
| 32.45 | 28.74 | 35.66 | 5.03 | 15.84 | 9.68 | 0.14 |
| 40 | 10 | 40 | 11.9 | 13.13 | 9.7 | 0.55 |
| 23.65 | 7.59 | 61.14 | 9.52 | 13.77 | 9.72 | 0.18 |
| 24.36 | 7.48 | 56.04 | 14.01 | 13.23 | 9.73 | 0.11 |
| 37.9 | 12.6 | 35.66 | 15.73 | 13.24 | 9.74 | 0.05 |
| 15.24 | 5.45 | 76.42 | 4.78 | 11.75 | 9.76 | 0.03 |
| 28.1 | 8.42 | 50.95 | 14.43 | 13.58 | 9.77 | 0.13 |
| 22.35 | 16.51 | 45.85 | 17.18 | 16.29 | 9.8 | 0.27 |
| 22.56 | 7.84 | 50.95 | 20.55 | 12.94 | 9.86 | 0.05 |
| 27.65 | 9.99 | 45.85 | 18.4 | 13.89 | 10.05 | 0.09 |
| 13.29 | 8.21 | 56.04 | 24.36 | 13.11 | 10.08 | 0.05 |
| 16 | 7 | 66.23 | 12.66 | 13.13 | 10.1 | 0.29 |
| 19 | 7 | 66.23 | 9.66 | 13.34 | 10.11 | 0.27 |
| 11.5 | 25 | 47.5 | 17.9 | 16.89 | 10.11 | 0.34 |
| 8 | 8 | 66.23 | 19.66 | 12.73 | 10.25 | 0.07 |
| 33.81 | 9.61 | 45.85 | 12.63 | 13.82 | 10.26 | 0.03 |
| 22.62 | 8.82 | 50.95 | 19.51 | 13.68 | 10.37 | 0.08 |
| 15.55 | 30.53 | 45.85 | 9.96 | 17.96 | 10.4 | 0.22 |
| 3 | 45 | 50 | 3.9 | 12.1 | 10.5 | 0.35 |
| 21.5 | 7.41 | 56.04 | 16.94 | 13.05 | 10.52 | 0.07 |
| 18.35 | 27.25 | 50.95 | 5.35 | 19.26 | 10.55 | 0.29 |
| 20.45 | 19.14 | 50.95 | 11.36 | 17.95 | 10.56 | 0.42 |
| 19.12 | 16.02 | 56.04 | 10.71 | 17.85 | 10.71 | 0.19 |
| 8.31 | 6.98 | 61.14 | 25.47 | 11.69 | 10.74 | 0.23 |
| 12 | 20.03 | 56.04 | 13.82 | 17.82 | 10.79 | 0.24 |
| 22 | 12 | 66.23 | 1.66 | 17.51 | 10.8 | n,d, |
| 11.15 | 8.5 | 61.14 | 21.11 | 13.48 | 10.82 | 0.19 |
| 24.06 | 26.13 | 40.76 | 10.95 | 16.88 | 10.88 | 0.3 |
| 25 | 9 | 66.23 | 1.66 | 15.48 | 10.9 | 0.14 |
| 9.9 | 28.14 | 56.04 | 7.82 | 18.48 | 10.95 | 0.26 |
| 11.17 | 25.48 | 50.95 | 14.3 | 17.59 | 11.01 | 0.16 |
| 5.26 | 11.19 | 71.33 | 14.12 | 13.48 | 11.03 | 0.16 |
| 19.19 | 17 | 56.04 | 9.67 | 18.2 | 11.05 | 0.29 |
| 23.46 | 10.7 | 50.95 | 16.79 | 14.92 | 11.08 | 0.25 |
| 11.23 | 21.97 | 50.95 | 17.75 | 17.06 | 11.11 | 0.07 |
| 12.2 | 27.64 | 35.66 | 26.39 | 14.36 | 11.19 | 0.22 |
| 8.44 | 9.53 | 66.23 | 17.69 | 13.93 | 11.22 | 0.1 |
| 6.82 | 14.17 | 76.42 | 4.49 | 16.17 | 11.33 | 0.38 |
| 9.01 | 8.8 | 66.23 | 17.85 | 13.61 | 11.39 | 0.24 |
| 10 | 15 | 66.23 | 10.66 | 17.14 | 11.45 | 0.49 |
| 12 | 13 | 66.23 | 10.66 | 16.86 | 11.45 | 0.21 |
| 9.06 | 16.44 | 56.04 | 20.35 | 15.97 | 11.49 | 0.1 |
| 20.6 | 7.18 | 66.23 | 7.89 | 13.61 | 11.52 | 0.08 |
| 30.21 | 11.88 | 45.85 | 13.96 | 14.93 | 11.54 | 0.07 |
| 6.79 | 20.43 | 56.04 | 18.63 | 15.71 | 11.55 | 0.16 |
| 9.64 | 16.24 | 66.23 | 9.79 | 17.45 | 11.56 | 0.23 |
| 10.61 | 18.22 | 50.95 | 22.12 | 16.13 | 11.57 | 0.29 |
| 12.34 | 16.16 | 56.04 | 17.36 | 16.85 | 11.58 | 0.06 |
| 14 | 18 | 66.23 | 3.66 | 19.32 | 11.6 | 0 |
| 23.34 | 7.8 | 56.04 | 14.72 | 13.48 | 11.63 | 0.14 |
| 18.07 | 18.85 | 56.04 | 8.93 | 18.64 | 11.64 | 0.05 |
| 4.24 | 25.75 | 66.23 | 5.67 | 14.94 | 11.7 | 0.3 |
| 16.4 | 17.41 | 50.95 | 17.14 | 17.04 | 11.75 | 0.1 |
| 18.13 | 15.35 | 56.04 | 12.38 | 17.5 | 11.79 | 0.08 |
| 16 | 16 | 66.23 | 3.66 | 18.97 | 11.85 | 0.21 |
| 13 | 13 | 66.23 | 9.66 | 17.09 | 11.9 | 0.14 |
| 10 | 10 | 66.23 | 15.66 | 14.68 | 12.05 | 0.07 |
| 15 | 40 | 45 | 1.9 | 18.22 | 12.07 | 0.81 |
| 15.66 | 23.52 | 45.85 | 16.86 | 17.11 | 12.15 | 0.22 |
| 7.06 | 14.74 | 76.42 | 3.67 | 16.53 | 12.23 | 0.13 |
| 5.08 | 15.5 | 71.33 | 9.99 | 14.77 | 12.28 | 0.13 |
| 7 | 12 | 66.23 | 16.66 | 14.61 | 12.35 | 0.21 |
| 11.04 | 15.68 | 61.14 | 14.03 | 17.1 | 12.47 | 0.17 |
| 11.51 | 13.37 | 66.23 | 10.79 | 16.91 | 12.63 | 0.15 |
| 4 | 25 | 66.23 | 6.66 | 14.5 | 12.65 | 0.21 |
| 15 | 15 | 55 | 16.9 | 16.79 | 12.9 | 0.53 |

Table B: Comparision of RMSE and R-squared values during the leave-one-out crossvalidation between neuralnet, nnet and RSNNS algorithm.

| **number of hidden units** | **RMSE** | | | | **R^2^** | | | |
| --- | --- | --- | --- | --- | --- | --- | --- | --- |
|  | **neuralnet: logistic** | **neuralnet: tanh** | **nnet** | **RSNNS** | **neuralnet: logistic** | **neuralnet: tanh** | **nnet** | **RSNNS** |
| 9 | 0.923 | 0.899 | 1.405 | 2.477 | 0.923 | 0.929 | 0.851 | 0.437 |
| 10 | 0.933 | 1.113 | 1.289 | 2.523 | 0.920 | 0.887 | 0.848 | 0.414 |
| 11 | 0.949 | 0.836 | 1.483 | 2.494 | 0.921 | 0.936 | 0.821 | 0.428 |
| 12 | 0.97 | 1.034 | 1.902 | 2.537 | 0.916 | 0.907 | 0.759 | 0.412 |

Table C: RMSE and R-squared for values between the activation functions logistics and tanh during leave-one-out cross validation for the neuralnet method.

| **number of hidden units** | **logistic** | | **tanh** | |
| --- | --- | --- | --- | --- |
|  | **RMSE** | **R-squared** | **RMSE** | **R-squared** |
| 1 | 2.108 | 0.588 | 2.285 | 0.517 |
| 2 | 1.293 | 0.846 | 1.498 | 0.798 |
| 3 | 1.051 | 0.903 | 1.013 | 0.907 |
| 4 | 0.877 | 0.930 | 0.946 | 0.918 |
| 5 | 0.935 | 0.920 | 0.885 | 0.929 |
| 6 | 0.942 | 0.919 | 0.804 | 0.940 |
| 7 | 1.080 | 0.900 | 0.876 | 0.930 |
| 8 | 0.994 | 0.912 | 1.007 | 0.914 |
| 9 | 0.923 | 0.923 | 0.899 | 0.929 |
| 10 | 0.933 | 0.920 | 1.113 | 0.887 |
| 11 | 0.949 | 0.921 | 0.836 | 0.936 |
| 12 | 0.970 | 0.916 | 1.034 | 0.907 |
| 13 | 0.847 | 0.934 | 1.245 | 0.859 |
| 14 | 0.878 | 0.929 | 0.960 | 0.917 |
| 15 | 1.000 | 0.913 | 1.064 | 0.902 |
| 16 | 0.924 | 0.924 | 1.157 | 0.889 |
| 17 | 0.995 | 0.914 | 1.139 | 0.894 |
| 18 | 1.004 | 0.908 | 1.107 | 0.899 |
| 19 | 0.960 | 0.919 | 1.036 | 0.906 |
| 20 | 1.020 | 0.910 | 1.086 | 0.905 |
| 21 | 1.109 | 0.893 | 1.250 | 0.868 |
| 22 | 1.087 | 0.899 | 1.252 | 0.869 |
| 23 | 0.983 | 0.915 | 0.923 | 0.925 |
| 24 | 0.982 | 0.912 | 1.004 | 0.911 |
| 25 | 0.924 | 0.925 | 1.471 | 0.818 |

R-Scripts A: R-script used to obtained the Table 1. The different activation function and number of hidden units are selected using "act.fct=" and "hidden = ".
